# Soluble egg antigen of *Schistosoma Haematobium *induces HCV replication in PBMC from patients with chronic HCV infection

**DOI:** 10.1186/1471-2334-6-91

**Published:** 2006-06-06

**Authors:** Mostafa K El-Awady, Samar S Youssef, Moataza H Omran, Ashraf A Tabll, Wael T El Garf, Ahmed M Salem

**Affiliations:** 1 Department of Biomedical Technology, National Research Center,, El tahrir St., Dokki, Cairo, Egypt; 2 Department of biochemistry, Ain Shams University, El Abassia Sq., Cairo, Egypt

## Abstract

**Background:**

This study was conducted to **e**xamine, *in vitro *, the effect of soluble egg antigen (SEA) of *S. haematobium* on intracellular HCV RNA load in peripheral mononuclear cells (PBMC) as well as on cell proliferation in patients with chronic HCV infection.

**Methods:**

PBMC from 26 patients with chronic HCV infection were cultured for 72 hours in presence and absence of 50 μg SEA/ml medium. Intracellular HCV RNA quantification of plus and minus strands was assessed before and after stimulation. PBMC from five healthy subjects were cultured for 7 days, flow cytometric analysis of DNA content was used to assess the mitogenic effect of SEA on PBMC proliferation compared to phytoheamaglutinine (PHA).

**Results:**

Quantification of the intracellular viral load showed increased copy number/cell of both or either viral strands after induction with SEA in 18 of 26 patients (69.2%) thus indicating stimulation of viral replication. Flow cytometric analysis showed that mean ± S.D. of percent values of cell proliferation was induced from 3.2 ± 1.5% in un-stimulated cells to 16.7 ± 2.5 % and 16.84 ± 1.7 % in cells stimulated with PHA and SEA respectively.

**Conclusion:**

the present study supports earlier reports on SEA proliferative activity on PBMC and provides a strong evidence that the higher morbidity observed in patients co-infected with schistosomiasis and HCV is related, at least in part, to direct stimulation of viral replication by SEA.

## Background

Hepatitis C Virus (HCV) is the major agent in non A non B hepatitis with serious complications ranging from chronic inflammatory disease to hepatic cirrhosis and end stage liver failure or hepatocellular carcinoma (HCC). It is estimated that 170 millions world wide are infected with HCV. Egypt has unusually high prevalence of hepatitis C resulting in high morbidity and mortality from liver disease. Approximately 20% of blood donors are seropositive for HCV antibodies [1-5]. Schistosomiasis is another hepatotropic infection with a major burden on Egyptian patient population particularly in rural societies[1,6,7]. Co-infections with *Schistosoma mansoni *were repeatedly shown to augment pathogenesis induced by HBV and HCV hepatitis [2,8,9]. Subjects co-infected with HCV accelerate advancement of liver disease to the chronicity of HCV infection, cirrhosis and hepatocellular carcinoma and high incidence of viral persistence [10]. However similar studies on *Schistosoma haematibium *derived SEA has been scarcely reported.

Several reports attempted to reveal the underlying mechanisms of the severity of co-infection. The majority of these have focused on the role of growth factor rich soluble egg antigen (SEA), in contrary to adult worm soluble antigens, on angiogenic processes and granuloma formation mediated by peripheral blood mononuclear cell (PBMC) [11] and endothelial cell proliferation [12] through distinct intracellular signaling pathway[13]. Such mitogenic activity of SEA was shown to be cell specific, dose dependent and involves up regulation of cell cycle engine promoting genes [14].

Although HCV titer was shown to be higher in patients co infected with HCV and Schistosomiasis than patients with HCV infection alone, direct data on the effect of SEA on intracellular HCV replication in PBMC derived from patients with chronic hepatitis C are yet unavailable. Thus the rational of this study is to examine, *in vitro *, the influence of SEA on intracellular HCV RNA load in PBMC from patients with chronic HCV infection. Careful study of cell proliferation response to SEA allows analysis of relative induction in intracellular viral load versus stimulation of cell proliferation.

## Methods

### Subjects

Twenty six patients were enrolled in this study. All patients had detectable HCV antibodies by third generation ELISA (Dia Sorin, Torino, Italy). Serum concentrations of HCV RNA varied from 29 × 10^3 ^to 3 × 10^6 ^. Twenty four patients had genotype 4 of HCV genome, while the remaining two had 1b. None of the patients received treatment for HCV. All the patients had undetectable levels of HBsAg, schistosoma and HIV antibodies. Five subjects served as controls for normal PBMC stimulation studies. None of the 5 control subjects had detectable sero markers for viral or parasitic infections.

### Methods

#### Effect of SEA on cell proliferation

A lyophilized preparation of the soluble fraction of mature live *S. haematobium *eggs was supplied by Theodore Bilharz Research Institute, Imbaba, Egypt.

Ten ml blood were collected from each normal control subject [approved by the medical ethical committee of the National research center (NRC)] and PBMC were separated from whole blood using Ficoll separating solution. Cells were washed with PBS three times. Mixtures were then centrifuged at 1600 rpm for 10 min to collect the washed cell pellet. After the last wash, cells were re-suspended in 1 ml RPMI-1640, supplemented with 10% FCS, counted and adjusted with RPMI to be 0.75 million cells/ml media. Cells were then plated into a 6 well plate at 2 million cells per well. A preliminary experiment was done to explore the optimum concentration of SEA that induces cell proliferation. Cells were treated with 10, 50, 100 and 150 μg/ml culture of SEA. Results obtained proved that 50 μg/ml culture is optimal (data not shown). To compare the stimulatory effect of SEA with phytoheamaglutinine (PHA), cells were stimulated with PHA at 5 μg/ml culture medium, other two wells were stimulated with SEA at 50 μg/ml medium, and the last two wells were left un-stimulated as control. Cells were cultured in a humidified atmosphere at 37°C, 5% CO_2 _for 7 days. Media were changed every 48 hours. After 7 days, cells were washed, permeablized with 0.1% triton X-100 solution (v/v) for 6 min at 4°C, then stained with 50 μg/ml propidium iodide (PI) as a DNA-specific fluorochrome for 30 min at 4°C in a dark place. DNA index analysis was performed on FACS Calibur flow cytometer equipped with logarithmic amplifiers.

#### Effect of SEA on HCV replication in PBMC

Fifteen ml of blood were withdrawn from each patient [approved by the medical ethical committee of (NRC)], five of them were collected on EDTA and were used to extract total cellular RNA from PBMC (to assess the presence of intracellular HCV strands in PBMC), the remaining ten ml blood were collected on heparin to separate PBMC using Ficoll for *in vitro *experiments. Cells were washed 5 times with PBS, then re-suspended in RPMI 1640 supplemented with 10% FCS at a concentration of 0.75 × 10^6^/ml, plated on a 6 well plate, and cultured at 37°C, 5% CO_2 _in absence or presence of SEA (50 μg/ml culture). After 72 hr, cells were counted (using haemocytometer), collected, washed seven times with PBS, then subjected to RNA extraction with guanidinium isothiocyanate according to Chomczynski and Sacchi [15].

#### Reverse transcription – polymerase chain reaction (RT-PCR)

Total cellular RNA was reverse transcribed to cDNA in 25 μl reaction mixture containing 20 U of AMV reverse transcriptase (promega, Madison, WI, USA), 200 μM of each dNTP, 25 pmoles of either antisense primer (1CH: 5' -GGT GCA CGG TCT ACG AGA CCT-3') for plus strand or sense primer (2CH: 5' -AAC TAC TGT CTT CAC GCA GAA -3') for minus strand. The first round PCR was done in a total volume of 50 μl containing 200 μM of each dNTP, 1 × reaction buffer (10 mM Tris-HCl pH 8.8, 1.5 mM MgCl2, 50 mM KCl and 0.1% triton X-100), 2 U Taq polymerase (Finnzyme, Finland), 50 pmoles from each primer (2 CH and P2: 5' -TGC TCA TGG TGC ACG GTC TA -3'). Amplification was performed by 35 cycles of thermal cycling, each consists of denaturation at 94°C for 1 min, annealing at 55°C for 1 min and extension at 72°C for 1 min followed by a final extension step for additional 10 min. The reaction was then cooled to 4°C. Twenty percent of the reaction was taken for a second amplification round (35 cycles) with the internal pair of primers (D1: 5'-CGC AGA AAG CGT CTA GCC AT -3' and D2: 5' -ACT CGG CTA GCA GTC TCG CG -3'). Cycling conditions on the thermal cycler were the same as the first round. Products of PCR were analyzed on 2% agarose gel electrophoresis and photographed.

#### Quantification of intracellular HCV RNA

Plus-strand RNA was transcribed *in vitro *(using sp7 primer and transcription *in vitro *kit, Promega, WI) using as template a cloned fragment containing the entire 5' UTR in PGEM-T plasmid (unpublished data). 5' UTR RNA was quantified spectrophotometrically and standard amounts (2 × 10^6 ^– 2 × 10^7 ^copies) were reverse transcribed and amplified using the method described above. Amplified products from standard concentrations of 5' UTR RNA and from infected PBMC were resolved on 2% agarose gel stained with ethidium bromide. Polaroid photographed gels were scanned and the intensity of the amplified bands were analyzed using Total Lab software (Phoretix, New Castle, UK). Number of copies of plus-strand RNA in each specimen was calculated on the standard curve. The later was made from serial dilutions of RNA prepared by *in vitro *transcription and the intensity units of scanned amplified bands using software units.

## Results

### Detection of HCV in circulating PBMC

Plus HCV RNA strand was detectable in sera and PBMC derived from all the 26 HCV patients. Both plus and minus RNA strands were detectable in PBMC of 21 patients, the remaining 5 patients had only plus strand detectable in their circulating PBMC (Table 1).

**Table 1:** Results of RT-PCR amplification of plus and minus RNA strands in circulating PBMC from 26 HCV positive patients.

**Patients**	**Age**	**Sex**	**Genotype**	**Serum Plus strand**	**PBMC Plus strand +ve**	**PBMC Minus strand**
**R.F.**	**35**	**♂**	**4**	+	+	+
**M.H.**	**46**	**♂**	**4**	+	+	+
**S.H.**	**45**	**♂**	**4**	+	+	+
**M.AA.**	**40**	**♂**	**4**	+	+	+
**809**	**37**	**♂**	**4**	+	+	-
**K.I.**	**30**	**♂**	**4**	+	+	+
**G.B.**	**37**	**♂**	**4**	+	+	+
**S.A.**	**36**	**♀**	**1b**	+	+	+
**F.AA.**	**42**	**♀**	**4**	+	+	+
**no 47**	**45**	**♂**	**4**	+	+	+
**A.N**	**37**	**♂**	**4**	+	+	+
**M.W.**	**40**	**♂**	**4**	+	+	+
**A.AZ.**	**37**	**♂**	**4**	+	+	-
**Y.AM.**	**42**	**♂**	**1b**	+	+	+
**R.M.**	**42**	**♂**	**4**	+	+	+
**no 51**	**53**	**♂**	**4**	+	+	+
**A.M.**	**47**	**♂**	**4**	+	+	+
**M.A.**	**42**	**♂**	**4**	+	+	+
**AH.AZ.**	**45**	**♂**	**4**	+	+	-
**A 730**	**47**	**♂**	**4**	+	+	+
**A.A.**	**43**	**♂**	**4**	+	+	+
**M.O.**	**45**	**♂**	**4**	+	+	+
**K.M.**	**48**	**♂**	**4**	+	+	+
**A.S.**	**38**	**♂**	**4**	+	+	+
**S.Y.**	**36**	**♂**	**4**	+	+	-
**S.K.**	**43**	**♀**	**4**	+	+	-

### Effect of SEA on PBMC proliferation

**Figure 1:**
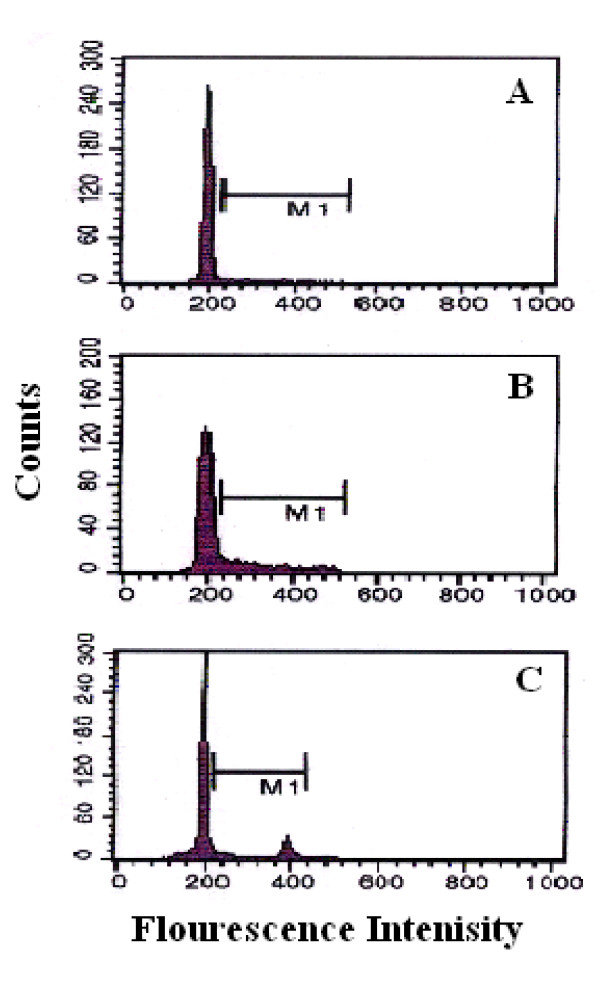
Flow cytometric analysis of cell proliferation (S+G2M) of PBMC. A) Normal un-stimulated PBMC, B) Normal PBMC stimulated with 5 μg/ml PHA, C) Normal PBMC stimulated with 50 μg/ml SEA after 7 days of culture.

Flow cytometric analysis showed that the mean ± S.D. of percent values of cell proliferation from 5 control un-stimulated cell cultures at day 7 was 3.2 ± 1.5 %. Stimulation of the 5 control cultures with PHA and SEA resulted in a significant (p < 0.01) increase in DNA index to 16.7 ± 2.5 % and 16.84 ± 1.7 % respectively (figure 1).

### Effect of SEA on cultured PBMC count from HCV patients

**Figure 2:**
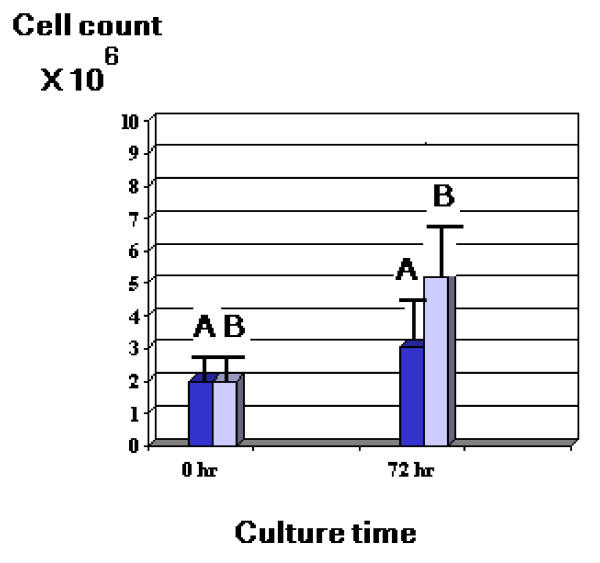
Mean ± S.D. value of cell counts of PBMC cultures from 26 HCV patients with (B) and without (A) SEA stimulation.

Stimulation of patients' PBMC in culture for 72 hours resulted in increased PBMC count in all cases ranging from 2 – 5 fold increase with a mean value of 4.26 ± 1.5 compared to non stimulated patients' cells that showed mean increase in cell count ranging from 1.3–2.3 fold only with a mean value of 1.75 ± 0.25 (Fig 2).

### Effect of SEA on HCV replication in PBMC in vitro

**Figure 3:**
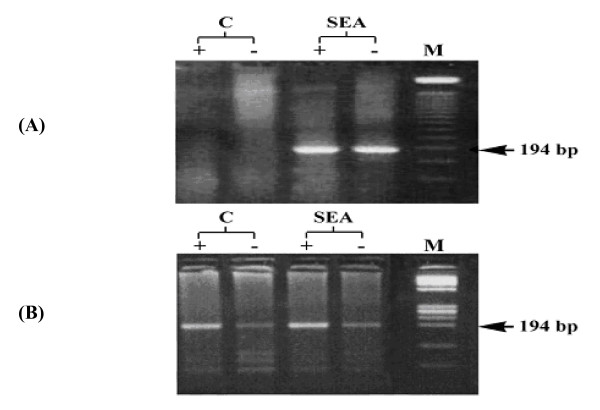
Results of PCR amplification of intracellular plus (+) and minus (-) RNA strands in PBMC derived from HCV patients before (C) and after stimulation with SEA in inducible (A) and non inducible (B) PBMC cultures. M is the molecular weight marker.

Quantification of the intracellular viral load (table 2) showed increased copy number/cell of both or either viral strands after stimulation with SEA in 18 of 26 patients (69.2%) thus indicating induction of viral replication (Fig 3, table 2). Five out of 21 (23.8%) patients lost preexisting viral strands upon culture. SEA stimulated cells of those patients showed restoration of either or both strands (Table 2).

**Table 2:** Effect of SEA on HCV replication in PBMC in vitro

**patients**	**Before stimulation (copies/cell)**		**After stimulation (copies/cell)**		**Induction**
	Plus	minus	Plus	minus	
R.F.	169	196	496	148	**I**
M.H.	146	93	UD	119	**I**
S.H.	UD	UD	64	64	**I**
M.AA.	125	UD	UD	UD	**NI**
809	73	64	79	64	**I**
K.I.	UD	UD	UD	122	**I**
G.B.	42	51	64	UD	**I**
S.A.	185	UD	234	UD	**I**
F.AA.	100	UD	UD	UD	**NI**
no 47	92	137	126	UD	**I**
A.N	UD	UD	UD	86	**I**
M.W.	100	100	100	105	**I**
A.AZ.	UD	64	UD	64	**NI**
Y.AM.	64	64	64	86	**I**
R.M.	UD	100	100	100	**NI**
no 51	264	264	235	228	**NI**
A.M.	UD	UD	UD	122	**I**
M.A.	24	UD	38	84	**I**
AH.AZ.	104	100	100	90	**NI**
A 730	345	404	100	346	**NI**
A.A.	73	73	104	100	**I**
M.O.	182	257	185	259	**I**
K.M.	73	73	73	79	**I**
A.S.	UD	UD	64	UD	**I**
S.Y.	UD	UD	UD	UD	**NI**
S.K.	UD	UD	UD	UD	**NI**

UD: undetected, I: induced, NI: non induced

## Discussion

In Egypt, viral hepatitis along with schistosomiasis is the major cause of chronic liver disease and liver cirrhosis [16-18]**. **The present study provides, on molecular bases, ample evidence that SEA treatment *in vitro *induces HCV replication and therefore may explain the higher disease severity in subjects co-infected with HCV and Schistosomiasis. The notion that intracellular HCV replication was induced *in vitro *by SEA in 69% of patients represents the first direct evidence on the role of this potent mitogen on HCV replication. It also suggests that individual host factors play distinct roles in determining viral response *in vitro *and perhaps the overall morbidity in patients co-infected with the two diseases. Several cellular proteins such as Elongation factor 3 (elfa 3), ribosomal proteins S5 and S9 and Eukaryotic translation initiation factor (eIf 3), the polypyrimidine tract binding protein (PTB), heterogenous nuclear ribonucleoprotein C (hnRNPC) and la antigen were shown to regulate translation of HCV polyprotein precursor and hence intracellular replication of viral genome [19-22]. Since PBMC have a major contribution into the circulatory pool of HCV load the results presented herein may explain why HCV titers are higher in patients with co-infection than those infected with HCV alone [23,24]. Furthermore we can not exclude similar role of SEA on hepatocytes harboring HCV genome in co-infected patients. Culturing the PBMC resulted in loss of both plus and minus RNA strands in 23.8% of cases. Interestingly SEA was capable of restoring either one or both strands in those cultures. This strengthens our hypothesis that SEA acts as a direct inducer of viral replication and makes it a valuable stimulatory reagent if future plans are considered to use short term PBMC cultures for antiviral screening. Recently, combinations of mitogens and/or cytokines were found to be able of stimulating synthesis of plus and minus RNA strands of HCV in PBMC and, in certain cases, affinity-purified T and B cells [25]. The exact mechanism whereby SEA stimulates HCV replication is not yet clear. A mechanism involving extracellular signaling of the growth factor components of SEA is perhaps implicated. In agreement with the current data, Kamal et al.,[23] concluded that co-occurrence of the two diseases was characterized by higher HCV RNA titers, histological activity, incidence of cirrhosis and hepatocellular carcinoma (HCC) compared to HCV infection alone. In the present study, the 5 fold increase in DNA index after 7 days and the observed 2.5 fold increase in PBMC count after 72 hour exposure to SEA indicate that SEA mediated cell proliferation involves increased rate of cellular DNA replication. In this regard both SEA and the commercial cellular mitogen PHA exerted similar magnitudes of DNA replication in the studied controls within 7 days of culture. Reports on other cell systems from our laboratory [14] and others [26,27] demonstrated that SEA associated cell proliferation involved up-regulation of cell cycle controlling genes such as peripheral cell nuclear antigen (PCNA) and B-cell translocation gene 1 (BTG1). Previous studies [28] reported that most soluble egg antigen fractions elicited *in vitro *granuloma reactions by PBMC of almost all *Schitosoma haematobium *infected patients and in vivo via endothelial cell proliferation [12,29] Conversely, separated soluble adult-worm antigens failed to stimulate PBMC of infected patients to form granulomas [28] suggesting that SEA contains unique factor (s) which are lacking in adult worm antigens. Furthermore, the role of SEA in PBMC proliferation has been well established as an initial step for angiogenesis and granuloma formation in Schistosomiasis patients by up-regulation of vascular endothelial cell growth factor. In HCC, the proliferative activity of vascular endothelial cells is suggested to play an important role in the positive regulation of tumor – associated vascular endothelial cell proliferation [30]. However, *in vitro *stimulatory effect of SEA on PBMC from chronic HCV patients was scarcely investigated. The significance of PBMC in chronic hepatitis C patients is based on the fact that PBMC represent a large extrahepatic reservoir for HCV replication. We and others have shown that PBMC from chronic HCV patients do not only contain HCV genomes but also function to maintain genomic replication [31-34] and to support viral core and envelope glycoprotein E1 expression in culture [34-36]. The two mitogens tested in the present study appeared not to have the same function on intracellular HCV titers indicating that different mechanisms of mitogen-viral interactions are involved. Induction of viral replication does not always correlate with increased cell proliferation, as SEA induced cell proliferation was notable in all patients even those whom intracellular HCV replication was not induced. Taken together the dual stimulatory function on both PBMC and viral copies per cell, the magnitude of total viral pool response in SEA stimulated culture is obviously larger than the currently presented values, in table 2, as viral copies per cell. The stimulatory function of SEA on replication of DNA viruses was reported as early as 1989 when Ishii et al.,[37] reported that SEA from *Schistosoma japonicum *induced Epstein Barr virus RNA in lymphoblastoid cells.

## Conclusion

The present study suggests that SEA possesses potent *in vitro *proliferative activity on PBMC and provides the first evidence that SEA directly stimulates HCV replication *in vitro *. This may explain, at least in part, the higher morbidity observed in patients co-infected with Schistosomiasis and HCV.

## Competing interests

The author(s) declare that they have no competing interests.

## Authors' contributions

MKE conceived the study, participated in data analysis and revised the manuscript. SSY designed the study, performed all culture steps, carried out molecular detection and quantification of HCV, analyzed the data and drafted the manuscript. MHO performed HCV genotyping, participated in design of the study and revision of the manuscript. AAT performed the flow cytometry experiments. WTE helped in culture work. AMS revised the manuscript carefully. All authors read and approved the final manuscript.

## Pre-publication history

The pre-publication history for this paper can be accessed here:


